# Altered Gut Microbiota and Predicted Immune Dysregulation in Early Childhood SARS-CoV-2 Infection

**DOI:** 10.3390/microorganisms13081879

**Published:** 2025-08-12

**Authors:** Dong Hyun Kim, Byung Ok Kwak, Ky Young Cho

**Affiliations:** 1 Department of Pediatrics, Inha University College of Medicine, 27, Inhang-ro, Jung-gu, Incheon 22332, Republic of Korea; id@inha.ac.kr; 2 Department of Pediatrics, Hallym University Kangnam Sacred Heart Hospital, 1 Singil-ro, Yeongdeungpo-gu, Seoul 07441, Republic of Korea

**Keywords:** microbiota, coronavirus disease 2019 (COVID-19), severe acute respiratory syndrome coronavirus 2 (SARS-CoV-2), early childhood, dysbiosis

## Abstract

The gut microbiome plays a key role in immune regulation. Young children experience rapid microbiome development, yet data on its alteration during severe acute respiratory syndrome coronavirus 2 (SARS-CoV-2) infection remain limited. This study aimed to characterize gut microbiome changes and immune-related pathway alterations in young children with coronavirus disease 2019 (COVID-19). Eighteen children under 2 years old with confirmed SARS-CoV-2 infection and seven healthy controls were enrolled between December 2021 and June 2022. Stool samples were analyzed using 16S rRNA gene sequencing. In children with COVID-19, the gut microbiome exhibited an increase in Bacteroidota and Bacillota, whereas Actinomycetota and Pseudomonadota were reduced, with higher abundances of *Bifidobacterium*, *Escherichia*, and *Streptococcus* and lower abundances of *Faecalibacterium*, *Clostridium*, and *Ruminococcus* compared with healthy controls. Children with COVID-19 exhibited reduced alpha diversity, indicating microbial dysbiosis, and significant differences in beta diversity compared with healthy controls. Predictive functional analysis revealed downregulation of key immune-related pathways, such as interleukin-17, NOD-like receptor, and Toll-like signaling, which may impact mucosal immunity and viral clearance in children with COVID-19. SARS-CoV-2 infection in early childhood is associated with gut dysbiosis and the suppression of key immune pathways. These findings highlight the potential long-term impact of early-life microbial disruptions on immune development.

## 1. Introduction

Coronavirus disease 2019 (COVID-19), caused by severe acute respiratory syndrome coronavirus 2 (SARS-CoV-2), was first reported in late 2019 and rapidly escalated to a global pandemic, as declared by the World Health Organization in March 2020 [1,2]. While COVID-19 primarily presents with respiratory symptoms, gastrointestinal manifestations, such as vomiting, diarrhea, and abdominal pain, are frequently observed in pediatric patients [3,4,5]. Emerging evidence suggests that the gut microbiome plays a pivotal role in immune system regulation and may influence the course of COVID-19 [6,7,8,9,10,11,12,13,14,15]. In adult patients with COVID-19, significant alterations in gut microbial composition—characterized by reduced microbial diversity, depletion of beneficial commensals, and enrichment of opportunistic pathogens—have been consistently observed, even in mild cases and after recovery [6,7,8,9,10]. However, studies in pediatric populations remain limited and yield inconsistent findings. These inconsistencies likely stem from variations in the study design, cohort age, symptom severity, and analysis methods [11,12,13,14,15].

Infants and toddlers experience rapid development of the gut microbiome, which strongly influences immune maturation. Because immune imprinting in early life may have long-term health implications, understanding the effect of SARS-CoV-2 infection on the gut microbiome in this age group is critical [12,16,17,18].

This study aimed to characterize the gut microbiome composition of children under 2 years old with mild to moderate COVID-19, compared with healthy controls, using 16S rRNA gene sequencing and predictive functional profiling.

## 2. Materials and Methods

### 2.1. Study Participants

This study enrolled 18 children under 2 years of age with confirmed COVID-19 who were admitted to Inha University Hospital and seven healthy children between December 2021 and June 2022. SARS-CoV-2 infection was confirmed using a reverse transcription polymerase chain reaction (PCR) assay performed on nasopharyngeal swabs. All children in the COVID-19 group experienced their first episode of SARS-CoV-2 infection, with no cases of reinfection included. Demographic and clinical data were collected through caregiver interviews and review of medical records, and were analyzed for each patient.

All seven healthy children had no known underlying medical conditions and were considered clinically healthy at the time of enrollment. They had no history of SARS-CoV-2 infection and exhibited no symptoms of COVID-19 at the time of stool sample collection. In addition, none had received probiotic or antibiotic therapy within the three months prior to sample collection.

### 2.2. Stool DNA Extraction, PCR Amplification, 16S rRNA Gene Sequencing, and Bioinformatics Analysis

Stool sample collection was explained upon admission, and samples were obtained at the first stool passage during hospitalization. No child had received antibiotics or probiotics prior to sample collection. Stool samples were collected from both healthy children and those with COVID-19, and were stored at −80 °C until DNA extraction. Microbial DNA was extracted from approximately 200 mg of fecal samples using the FastDNA™ SPIN Kit for Soil (MP Biomedicals, Solon, OH, USA), according to the manufacturer’s instructions. Then, 16S rRNA gene sequencing was conducted on the V3–V4 regions by a commercial service provider (CJ Bioscience., Seoul, Republic of Korea) using the Illumina MiSeq platform (Illumina, San Diego, CA, USA) [19]. Taxonomic profiling was performed using EzBioCloud’s MTP 16S service with the PKSSU4.0 reference database (CJ Bioscience, Seoul, Republic of Korea). Operational taxonomic units (OTU) read counts were imported into R version 4.0.3 (R Foundation for Statistical Computing, Vienna, Austria) for statistical analysis. OTU counts were rarefied to 12,746 reads (minimum among samples). Linear discriminant analysis effect size (LEfSe) was used to identify differentially abundant taxa between children with COVID-19 and healthy controls. Alpha diversity indices, including Shannon diversity and species richness, were calculated. Beta diversity was assessed using the non-metric multidimensional scaling of Bray−Curtis index, with principal coordinate analysis for visualization. Permutational multivariate analysis of variance (PERMANOVA) was performed to compare the dissimilarities between two groups. Functional metabolic pathway enrichment analysis was conducted using data from the Kyoto Encyclopedia of Genes and Genomes (KEGG) database. Significant differences in KEGG predictions between the two groups were identified using LEfSe, with a significance threshold (α) of 0.01 and LDA score of 3.0. For taxonomic assignment, microbial community profiling, and functional prediction based on 16S rRNA gene profiles, we utilized EzBioCloud (www.ezbiocloud.net) (accessed on 30 March 2024), a comprehensive web-based platform that includes modules for inferred functional profiling using KEGG orthology.

### 2.3. Statistical Analysis

Normally distributed data were analyzed using the *t*-test and are presented as means ± standard deviations. In contrast, non-normally distributed data were analyzed using the Mann−Whitney U test and are presented as medians with interquartile ranges. Categorical variables were analyzed using the chi-square test or Fisher’s exact test and are presented as frequencies and percentages.

Statistical significance was set at *p* < 0.05. Differences in alpha diversity and relative abundances of bacterial taxa between groups were analyzed using the Wilcoxon rank-sum test, with *p*-values adjusted for multiple testing using the Benjamini−Hochberg procedure to control the false discovery rate (FDR).

### 2.4. Ethics Statements

The study was conducted in accordance with the Declaration of Helsinki, and approved by the Institutional Review Board (IRB) of Inha University Hospital (IRB No. IUH-2021-07-008) on 12 July 2021. Written informed consent was obtained from the parents or legal guardians of all participants.

## 3. Results

### 3.1. Participant Characteristics

The study included 18 children with confirmed SARS-CoV-2 infection and seven healthy children. The demographic and clinical characteristics of the participants are summarized in Table 1. The median age of children with COVID-19 was 4.5 months (range: 25 days–16 months), and 10 (55.6%) were male. Most of the children with COVID-19 were fed infant formula (77.8%), whereas the rest were on weaning foods (16.7%) or a regular diet (5.5%). Among children with COVID-19, fever (83.3%) was the most common symptom, followed by cough (80.0%), sputum (30.0%), nasal obstruction (30.0%), and rhinorrhea (20.0%). Notably, none of the children reported gastrointestinal symptoms, such as vomiting or diarrhea. The median duration from symptom onset to stool sample collection was 3 days (range: 0–9 days). Pneumonia was suspected in 12 (66.7%) children, and coinfections were identified in five (27.8%) cases. The identified pathogens included rhinovirus (3 cases, 16.7%), human coronavirus 229E (1 case, 5.5%), and respiratory syncytial virus B (1 case, 5.5%). During hospitalization, five (27.8%) children received antibiotic therapy, while none were administered antiviral agents or corticosteroids. None of the children with COVID-19 had received antibiotics prior to admission or stool sample collection.

The healthy control group comprised five boys (71.4%) and two girls (28.6%), with a median age of 30 months (range: 3–76 months). None of the healthy children had comorbidities or coinfections.

### 3.2. Gut Microbiome Composition Analysis

**Figure 1:**
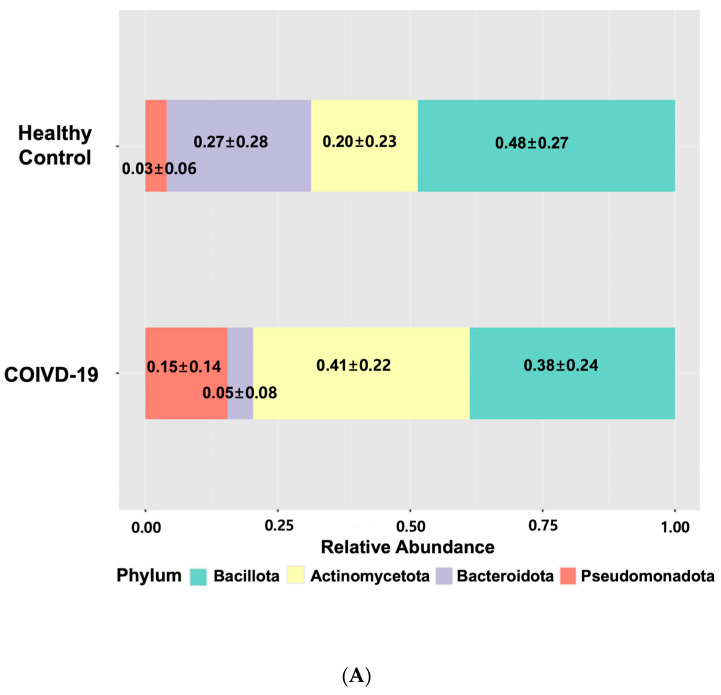

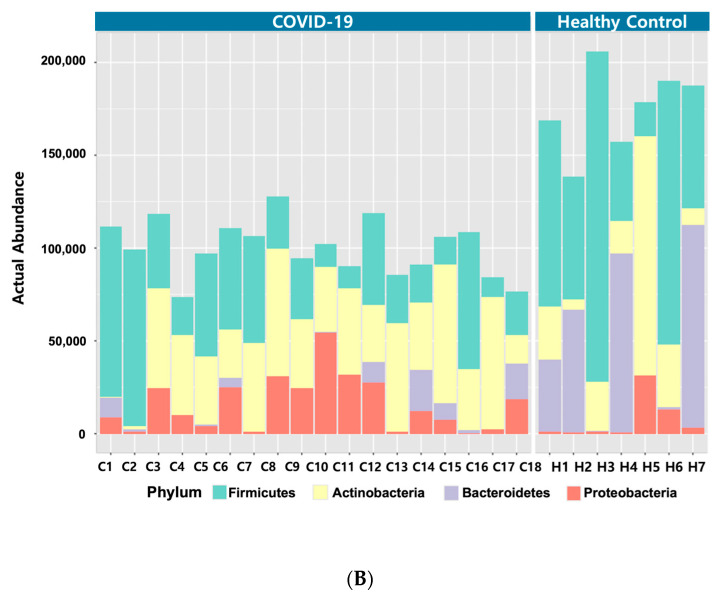
The composition of the gut microbiome at the phylum level in healthy children and those with COVID-19. (**A**) Relative abundance ratio of bacteria at the phylum level in healthy children and those with COVID-19. Values are mean ± SD. (**B**) The actual abundance of gut microbiome at phylum level in healthy children and those with COVID-19. COVID-19, coronavirus disease 2019.

**Figure 2:**
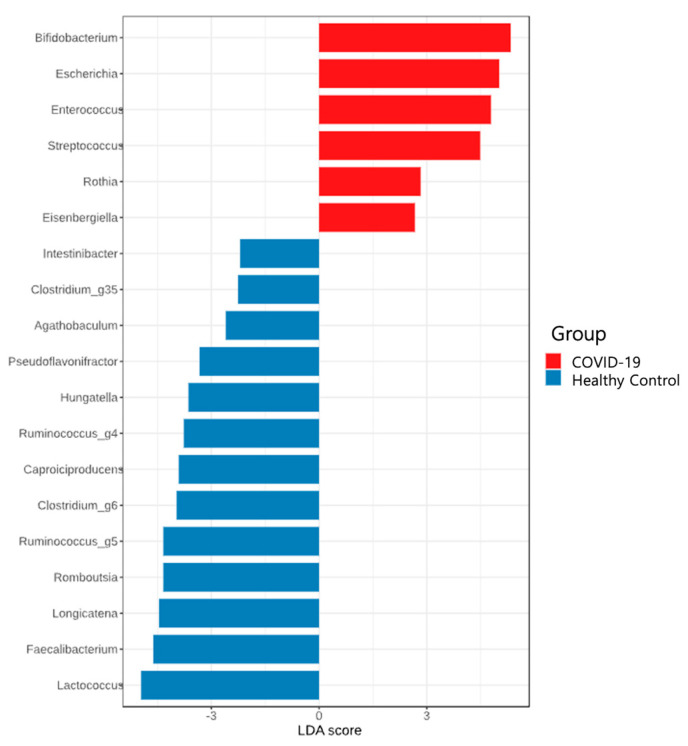
Relative abundance ratio of bacteria at the genus level in healthy children and those with COVID-19. LEfSe analysis showing bacterial taxa with the greatest differences in abundance between the children with COVID-19 and healthy children. LDA, linear discriminant analysis; LEfSe, Linear discriminant analysis effect size; COVID-19, coronavirus disease 2019.

To characterize the gut microbiome composition in children, 16S rRNA gene sequencing was performed on fecal samples from 18 children with COVID-19 and seven healthy children. Compared with the healthy children, those with COVID-19 exhibited a decrease in the relative abundance of bacterial phyla Bacteroidota and Bacillota and a significant increase in the abundances of Actinomycetota and Pseudomonadota (Figure 1A,B, and Figure S1). LEfSe analysis identified multiple differentially abundant taxa between the two groups across various taxonomic levels. Using an LDA score > 3.0 for feature selection, several bacterial genera were different between children with COVID-19 and healthy controls. Specifically, higher relative abundance of the genera *Bifidobacterium*, *Escherichia*, *Enterococcus*, and *Streptococcus* were observed in children with COVID-19, whereas lower relative abundance of *Lactococcus*, *Faecalibacterium*, *Longicatena*, *Romboutsia*, *Ruminococcus_g^5^*, *Clostridium_g^6^*, *Caproiciproducens*, *Ruminococcus_g^4^*, *Hungatella*, and *Pseudoflavonifractor* were identified in the healthy children (Figure 2).

**Figure 3:**
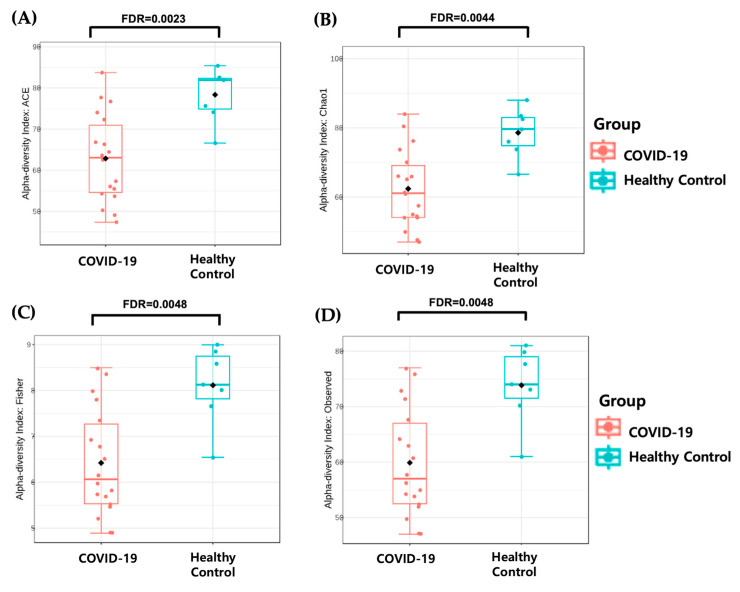
Alpha diversity analysis between children with COVID-19 and healthy children by ACE (**A**), Chao1 (**B**), Fisher (**C**), and observed_OTUs index (**D**). The box plots denote the interquartile range, 25th to 75th percentile. The median values are shown as a line within the box. Statistical significance was assessed using Mann–Whitney U test. The multi-testing was adjusted using the Benjamini–Hochberg procedure (FDR). COVID-19, coronavirus disease 2019; OTU, operational taxonomic units; FDR, false discovery rate.

**Figure 4:**
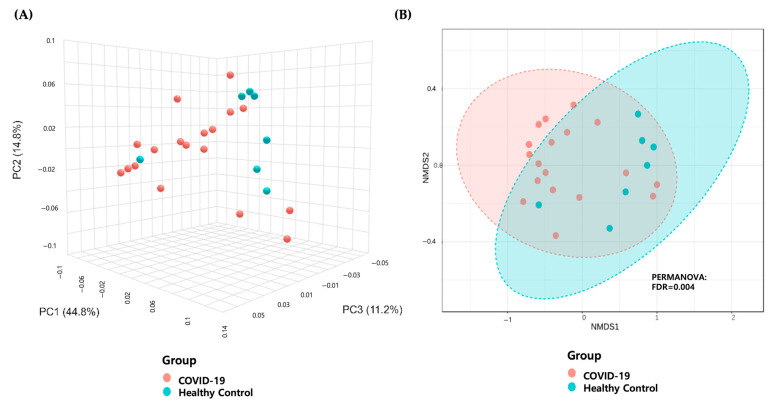
Beta diversity analysis between children with COVID-19 and healthy children. (**A**) Principal coordinate analysis based on the Bray−Curtis distance in relative microbiome profiles in each group. Each dot represents one sample. Red circles represent the COVID-19 group, and blue circles represent the healthy control group. (**B**) Beta diversity analysis between COVID-19 children and healthy children by NMDS of Bray−Curtis distance. Each dot represents one sample, and the ellipses represent groups. Groups were compared using the PERMANOVA method. COVID-19, coronavirus disease 2019; FDR, false discovery rate; NMDS, non-metric multidimensional scaling; PERMANOVA, permutational multivariate analysis of variance.

A significant difference in alpha diversity was observed between the two groups, as indicated by the Abundance-based Coverage Estimator (ACE, FDR = 0.0023), Chao1 richness estimator (Chao1, FDR = 0.0044), Fisher (FDR = 0.0048), and observed_OTUs (FDR = 0.0048) indices (Figure 3). In addition, beta diversity analysis, based on the Bray−Curtis distance, revealed significant clustering of samples between the children with COVID-19 and the healthy controls, as assessed using the PERMANOVA method (Figure 4).

### 3.3. Functional Profile Analysis of the Gut Microbiome

**Figure 5:**
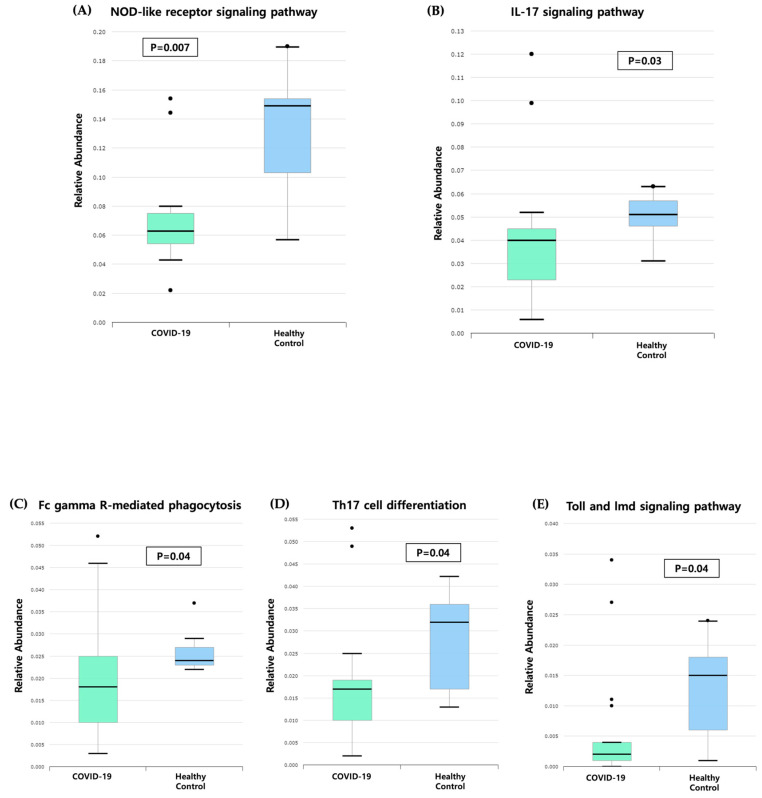
Functional profile analysis of the gut microbiome in the children with COVID-19 and the healthy children. The box plots denote the interquartile range, 25th to 75th percentile. The median values are shown as a line within the box. COVID-19, coronavirus disease 2019; IL, interleukin.

The functional profile of the gut microbiome in healthy children and those with COVID-19 was analyzed (Figure 5). Several immune-related pathways, including the NOD-like receptor signaling pathway, interleukin-17 (IL-17) signaling pathway, Fc gamma R-mediated phagocytosis, Th17 cell differentiation, and Toll and Imd signaling pathways, were significantly downregulated in the children with COVID-19 compared with the healthy controls.

## 4. Discussion

This study investigated alterations in the gut microbiome of children with COVID-19. An increased relative abundance of the phyla Pseudomonadota and Actinomycetota and a decreased abundance of Bacteroidota and Bacillota were observed in the children with COVID-19. At the genus level, the relative abundances of *Bifidobacterium*, *Escherichia*, *Enterococcus*, and *Streptococcus* were higher, whereas *Lactococcus*, *Faecalibacterium*, *Longicatena*, *Romboutsia*, *Ruminococcus_g^5^*, *Clostridium_g^6^*, *Caproiciproducens*, *Ruminococcus_g^4^*, *Hungatella*, and *Pseudoflavonifractor* were significantly lower in the children with COVID-19 than in the healthy controls. We observed significantly reduced alpha diversity and distinct beta diversity clustering in the children with COVID-19 compared with the healthy children.

The strength of this study lies in its focus on gut microbiome alterations in children with SARS-CoV-2 infection during early childhood—a population that has been underrepresented in prior studies due to typically mild symptoms, infrequent testing, and sample collection challenges. The developing gut microbiome in children plays a crucial role in immune regulation and inflammatory responses, emphasizing the need to investigate microbiome−viral interactions and their impact on disease severity [20,21]. The gut microbiota undergoes significant compositional and functional changes throughout life, with the most rapid and dynamic development occurring in early childhood, shaping both the immune and metabolic systems [18]. During infancy, the microbiome is highly influenced by genetic and environmental factors, including infections and antibiotic use [11,16]. Disruptions in this microbiome development have been linked to an increased risk of health issues later in childhood and adulthood [11].

Angiotensin-converting enzyme 2 (ACE2), which serves as the SARS-CoV-2 receptor, is highly expressed in multiple organs, including the lungs and gut. ACE2 expression in the small intestine is 2.5-fold higher in children than in adults, potentially explaining the higher prevalence of gastrointestinal symptoms in pediatric COVID-19 cases [2,22]. Differences in ACE2 expression and its interaction with the gut microbiota may contribute to the varied clinical outcomes between children and adults [20]. In this study, no children presented with gastrointestinal symptoms, which may be attributed to the small sample size, the inclusion of only mild to moderate COVID-19 cases, and the relatively short duration between symptom onset and specimen collection.

Previous studies have reported gut microbiome alterations in adult patients with COVID-19, with dysbiosis correlating with disease severity [6,7,8,9,10]. Even asymptomatic and mild cases exhibited gut microbiome disruptions, whereas more severe cases showed a greater depletion of beneficial taxa and an increase in inflammatory cytokines [6,7,8,20]. Dysbiosis persisted even after viral clearance and the resolution of respiratory symptoms [8].

In pediatric COVID-19, limited studies suggest similar microbiome disruptions, although findings vary across cohorts and methodologies [11,12,13,14,15]. Xu et al. [11] reported an increase in Bacteroidota and Bacillota with a decrease in Pseudomonadota in children during early COVID-19. Opportunistic pathogens, such as *Pseudomonas*, *Herbaspirillum*, and *Burkholderia*, also showed an increase in abundance in children with COVID-19 compared with healthy children [11]. Even asymptomatic infants exhibited decreased anti-inflammatory bacteria, such as *Bifidobacterium bifidum* and *Akkermansia muciniphila*, despite the lack of difference in alpha and beta diversity compared with uninfected infants [12]. Romani et al. [14] found that children with COVID-19 had a distinct gut microbiome profile, characterized by lower alpha diversity and increased opportunistic pathogens (e.g., Fusobacteria and Bacteroidota), with a depletion of Actinomycetota and Verrucomicrobia. Disease severity in pediatric COVID-19 correlated with reduced microbiome diversity and depletion of anti-inflammatory bacteria [12,14]. Suskun et al. [13] reported no significant difference in Bacteroidota abundance between children with and without COVID-19, although children with multisystem inflammatory syndrome (MIS-C) exhibited a higher Bacteroidota-to-Bacillota ratio. In addition, they noted a decrease in *Faecalibacterium prausnitzii* (a key anti-inflammatory species) and an increase in *Eggerthella lenta* (linked to Th17 activation and autoimmunity) in children with COVID-19. Further, Wang et al. [15] found that children infected with the Omicron variant had a lower abundance of Actinomycetota and Bacillota but a higher abundance of Bacteroidota, Pseudomonadota, and Verrucomicrobiota. Their gut microbiome was enriched in *Prevotella*, *Lachnoclostridium*, *Escherichia-Shigella*, and *Bacteroides*, whereas *Blautia*, *Bifidobacterium*, *Fusicatenibacter*, *Streptococcus*, and *Romboutsia* were depleted compared with healthy controls.

Consistent with previous findings, our study showed an increased abundance of opportunistic pathogens, including *Escherichia*, *Enterococcus*, and *Streptococcus*, and a decrease in commensal bacteria with anti-inflammatory properties, such as *Lactococcus*, *Faecalibacterium*, and *Ruminococcus*. These microbiome disruptions during infancy may have long-term health implications, necessitating further research into their potential impact on immune and inflammatory responses [11,12,20].

Our study identified the downregulation of immune pathways in children with COVID-19, including the NOD-like receptor signaling pathway, IL-17 signaling pathway, Fc gamma R-mediated phagocytosis, Th17 cell differentiation, and Toll and Imd signaling pathway. NOD-like receptor signaling, essential for intracellular pathogen recognition and inflammasome activation, has been associated with disease severity and poor clinical outcomes in patients with COVID-19 [23]. Similarly, the IL-17, a pro-inflammatory cytokine primarily produced by Th17 cells, plays a dual role in COVID-19 by contributing to mucosal antiviral defense and neutrophil recruitment while also promoting pathological inflammation when excessively activated [24]. The Fc gamma R-mediated phagocytosis facilitates the clearance of opsonized pathogens, but may also drive hyperinflammation through excessive activation [25]. In addition, Th17 cells play an important role in the pathogenesis of COVID-19, not only by activating cytokine cascade but also by inducing Th2 responses, inhibiting Th1 differentiation and suppressing Treg cells [26]. Failure to maintain the Th17 lineage in the gut is associated with microbial translocation that contributes to dissemination of the virus [27]. Toll and Imd signaling pathways are essential for the recognition of viral particles and activation of innate immune responses, leading to the secretion of pro-inflammatory cytokines such as IL-1, IL-6, and tumor necrosis factor-α, and type 1 interferons. Suppression of these pathways may impair pathogen recognition and antiviral defense, potentially allowing SARS-CoV-2 to evade immune detection. Conversely, excessive activation may trigger hyperinflammatory responses, contributing to immunopathology [28,29]. These findings suggest that alteration in the microbiome may affect the development and training of the immune system, highlighting the need for further research into targeted immunomodulatory therapies to restore immune balance. The immune-related signaling pathways described in this study are derived from microbial functional predictions based on 16S rRNA sequencing data and KEGG orthology mapping. These predictions represent the potential microbial contribution to host immune interactions, but do not reflect direct measurements of host cytokine or protein expression.

A major strength of this study is its focus on early childhood, a critical window for gut microbiome and immune system development, which remains understudied in the context of COVID-19. By combining taxonomic profiling with functional analysis of immune-related pathways, this study provides novel insights distinct from adult data and highlights age-specific host microbiome interactions.

This study has several limitations. First, since stool samples were collected at a single time point during hospitalization, it remains unclear whether the observed microbiome alterations preceded or resulted from COVID-19-related inflammation. Second, this study focused on mild-to-moderate COVID-19 cases in infants, excluding children with MIS-C or severe disease, who are known to exhibit distinct microbiome patterns and gastrointestinal symptoms [30]. Third, various external factors, such as diet, mode of delivery, breastfeeding, antibiotic use, and lifestyle changes during the pandemic, may have influenced the microbiome composition, but were not fully accounted for in this study [31]. Fourth, this study did not encompass all the pandemic periods, limiting its applicability to different SARS-CoV-2 variants. In addition, the limited clinical data available for the healthy control group and the small sample size may have restricted more detailed and statistically robust correlation analyses. Future longitudinal, multicenter studies with larger cohorts are needed to better understand the causal relationship between microbiome alterations and disease severity in pediatric COVID-19. Additionally, the absence of direct host immune data, such as cytokine or mRNA expression, is a limitation of this study, and future research will aim to validate the inferred microbial functions by incorporating host-derived transcriptomic or proteomic analyses.

## 5. Conclusions

Our findings demonstrate that SARS-CoV-2 infection in early childhood was associated with distinct alterations in the gut microbiome, including reduced diversity and immune-related functional changes. Given the critical role of the gut microbiome in immune development, understanding these changes is essential for evaluating potential long-term health implications. Future longitudinal, multicenter studies with large cohorts are needed to elucidate the role of the gut microbiome in pediatric COVID-19 and its impact on immune function, disease progression, and long-term health outcomes.

## Figures and Tables

**Table 1:** Demographic and clinical characteristics of participants.

	Children with COVID-19 (N = 18)	Healthy Children (N = 7)
Age (months), median (min–max)	4.5 (0.8–16)	30 (3–76)
Male, N (%)	10 (55.6)	5 (71.4)
Gestational age (weeks)	39	38
Birth weight (kg)	3.3	3.0
Mode of delivery, N (%)		
Vaginal delivery	5 (27.8)	1 (14.3)
Cesarean section	4 (22.2)	5 (71.4)
Unknown	9 (50.0)	1 (14.3)
Diet, N (%)		
Infant formula	14 (77.8)	2 (28.6)
Weaning	3 (16.7)	1 (14.3)
Regular diet	1 (5.5)	4 (57.1)
Duration between symptom onset and specimen collection, median (min–max)	3 (0–9)	-
Symptoms at admission, N (%)		-
Fever	15 (83.3)	-
Respiratory symptoms	10 (55.6)	-
Cough	8 (80.0)	-
Sputum	3 (30.0)	-
Nasal obstruction	3 (30.0)	-
Rhinorrhea	2 (20.0)	-
Gastrointestinal symptoms	0 (0.0)	-
Laboratory results, median		-
WBC (10^3^/μL)	6710	-
ESR (mg/dL)	11	-
CRP (mg/dL)	0.13	-
Procalcitonin (ng/mL)	0.12	-
AST (IU/L)	40.5	-
ALT (IU/L)	19.5	-
LDH	285	-
IL-6	11.6	-
Radiological findings		-
Suggestive of pneumonia, N (%)	12 (66.7)	-
Coinfection, N (%)	5 (27.7)	-
hCoV 229E	1 (5.5)	-
Rhinovirus	3 (16.7)	-
RSV B	1 (5.5)	-
Antibiotic use, N (%)	5 (27.8)	-

ALT, alanine transaminase; AST, aspartate transaminase; COVID-19, coronavirus disease 2019; CRP, C-reactive protein; ESR, erythrocyte sedimentation rate; hCoV 229E, human coronavirus 229E; IL, interleukin; LDH, lactate dehydrogenase; RSV B, respiratory syncytial virus B; WBC, white blood cells.

## Data Availability

The data are not publicly available due to privacy and ethical restrictions.

## Supplementary Materials

The following supporting information can be downloaded at: https://www.mdpi.com/article/10.3390/microorganisms13081879/s1, Supplementary Figure S1. Group-wise comparison of phylum level gut microbiota relative abundance. The box plots denote the interquartile range, 25th to 75th percentile. The median values are shown as a line within the box. Statistical significance was assessed using Mann–Whitney U test.
