# Supplementary figures and images for: Altered Gut Microbiota and Predicted Immune Dysregulation in Early Childhood SARS-CoV-2 Infection
Source: Microorganisms. 2025 Aug 12;13(8):1879. doi: 10.3390/microorganisms13081879 (PMC12388501; doi:10.3390/microorganisms13081879)

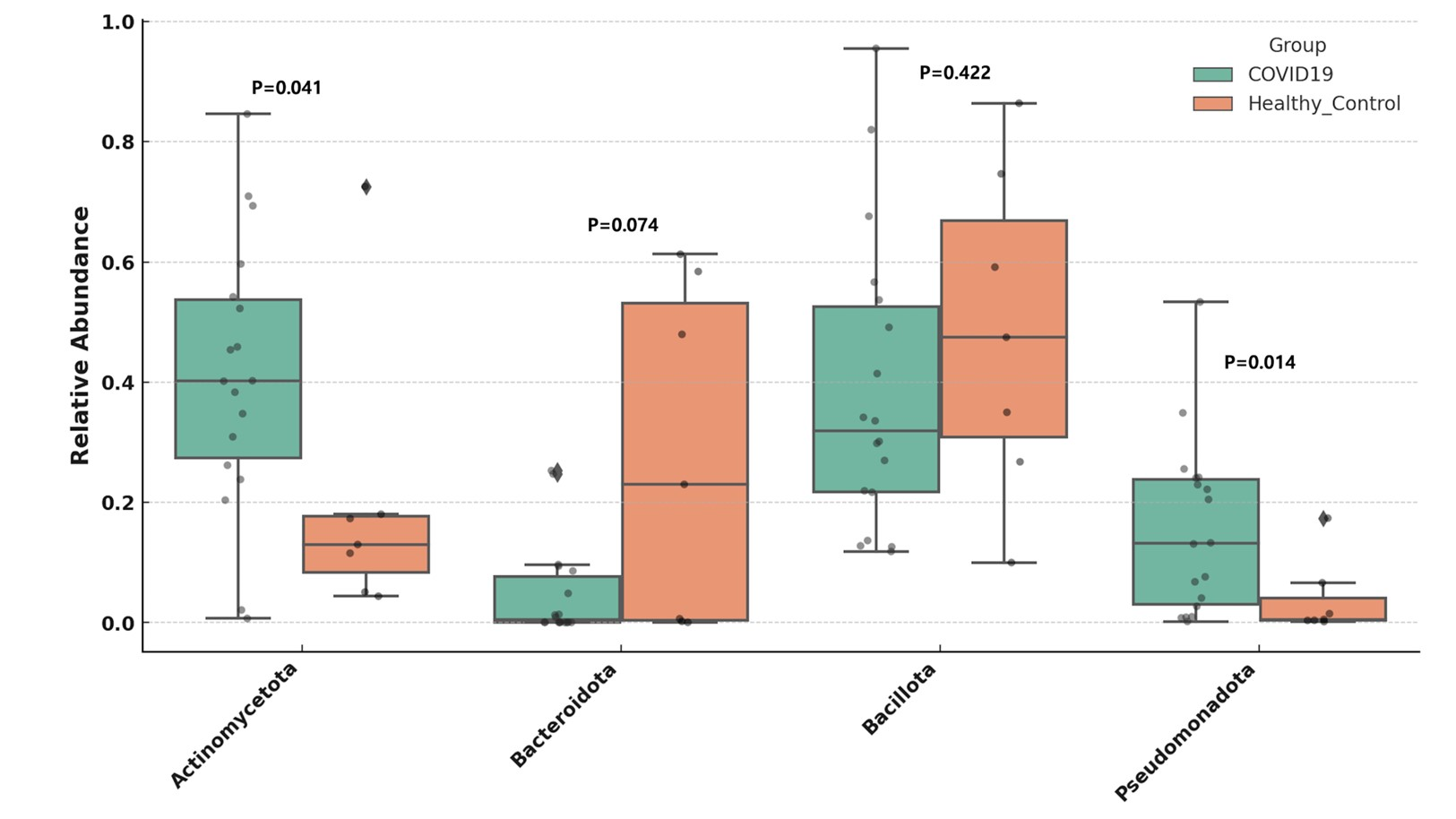

Supplement: Supplementary file 1 [file microorganisms-13-01879-s001.zip › microorganisms-3747831-supplementary Figure S1.tiff]
